# Assessing and enhancing the circularity and sustainability of emergency hospital shelters

**DOI:** 10.1111/disa.12670

**Published:** 2024-12-04

**Authors:** Eefje Hendriks, Joop de Zwart, Alexander Compeer, Julia Gospodinova

**Affiliations:** ^1^ Centre of Expertise Safety and Resilience Avans University of Applied Sciences, the Netherlands, and Department of Applied Earth Sciences, Faculty of Geo‐information Science and Earth Observation, University of Twente the Netherlands; ^2^ Centre of Expertise Safety and Resilience Avans University of Applied Sciences the Netherlands; ^3^ Centre of Expertise Biobased Economy Avans University of Applied Sciences the Netherlands; ^4^ Independent Researcher the Netherlands

**Keywords:** circularity, climate change mitigation, decision‐support tool, emergency hospital shelter, humanitarian, sustainability

## Abstract

Leading humanitarian organisations strive to enhance the sustainability of their aid to avoid negative impacts on the environment, economy, and society, particularly in low‐resource areas. This study explores how the circularity and sustainability of emergency hospital shelters can be assessed using literature, expert interviews, co‐creation and design sessions, and pilot testing. The approach combines a qualitative circularity checklist with a quantitative environmental impact assessment, providing valuable input for informed decision‐making during procurement and design. The findings reveal that existing buildings are commonly repurposed before importing emergency hospital shelters—finite virgin materials are primarily used for the production of new shelters—and there is a lack of data with which to reflect on end‐of‐life scenarios. The study recommends enhanced monitoring of the shelter lifecycle through data collection as an input for continuous improvement procedures of design and supply. Crucial are extended stakeholder responsibilities for the entire lifecycle and sector‐wide adoption of circularity and sustainability ambitions, mainstreaming approaches and showcasing benefits.

**Practitioner points**

1. Develop an organisational strategy for sustainability and circularity.
2. Collect data to track and measure the circularity of the full emergency hospital shelter life cycle.
3. Mainstream values and a mindset about circularity in the humanitarian sector for better results.

## 1 INTRODUCTION

Already vulnerable populations are disproportionately affected by prolonged and increasingly severe crises and more intense, frequent, and unpredictable weather conditions caused by climate change (Pörtner et al., 2023). Extended and severe humanitarian crises lead to higher emissions from humanitarian aid, worsening the climate crises and contributing to the need for long‐term assistance. This goes against the humanitarian principles of ‘doing no harm’ (UN, 1991, 2004). Humanitarian and governmental actors recognise their role and seek to reduce their environmental impact to minimise the negative influence on the health and well‐being of people, especially those living in resource‐constrained areas. Therefore, in line with the Sustainability Development Goals (SDGs) of the United Nations (UN), more than 400 humanitarian organisations have signed *The Climate and Environment Charter for Humanitarian Organizations* (ICRC and IFRC, 2021). Jointly they call for environmental guidelines to make more sustainable process, product, and procurement choices and thereby reduce their carbon footprint (UNHCR, 2015; Pirjevec, 2021; UN OCHA, 2021). Yet, it remains challenging to operationalise these ambitions. This study explores pathways to assess and enhance circularity and sustainability, taking humanitarian emergency hospital shelters as an example.

The global need for humanitarian assistance is increasing, with an estimated 406.6 million people in need in 2022, according to the *Global Humanitarian Assistance Report 2023* (Development Initiatives, 2023). The costs of humanitarian assistance are already high and mounting: USD 46.9 billion was spent in 2022 alone, a 25 per cent increase compared to previous years (Development Initiatives, 2023). Simultaneously, the estimated greenhouse gas (GHG) emissions related to humanitarian assistance are growing from approximately 18.5 Mt CO_2_e (megatons of carbon dioxide equivalent) in 2019 to approximately 35.3 Mt CO_2_e in 2022 (Godefroy et al., 2023).

Specifically, changes in procurement practices can considerably reduce emissions, as 75 per cent of the humanitarian sector's emissions can be allocated to purchasing goods and services and cash‐based interventions (Godefroy et al., 2023). Procurement is also crucial for achieving a circular economy, commonly accounting for 50 per cent of businesses' total product and service costs (Hald, Wiik, and Larssen, 2021). These numbers indicate the potential impact of environmentally‐friendly procurement guidelines on the planet, people, and prosperity. However, humanitarian funding notoriously falls short of meeting aid demands, with just 58 per cent of global funding requirements met in 2022 (Development Initiatives, 2023). Consequently, to scale up the scope of humanitarian aid, the prices of relief products and delivery times are often leading decision criteria in procurement, often overshadowing environmental criteria (Alshawawreh et al., 2020; Logistics Cluster, 2023b).

In the emergency phase, the procurement of shelters is unavoidable for humanitarian organisations in most crises and is therefore an important opportunity to enhance circularity and sustainability. Shelter and settlement assistance can cause many environmental issues on a local or global level, such as deforestation, GHG emissions, and plastic pollution (Brangeon and Crowley, 2020; Pirjevec, 2021). While shelters can take many forms, emergency shelters depend on rapid and efficient distribution, often in areas with limited access. Before deployment to areas in need, the production process and logistics of prefabricated emergency shelters require resources and energy which can contribute to environmental pollution. In terms of GHG emissions, the Emergency Shelter and Non‐Food Items cluster amounted to approximately 1.4 Mt CO_2_e in 2022 (Godefroy et al., 2023). To put this in perspective, this is roughly comparable to the emissions of 200,000 world citizens, based on a normalised average emission of eight tons per person per year (Sleeswijk et al., 2008). In addition, despite the relatively short lifespan of emergency shelters of several months, a clear strategy for their end‐of‐life phase is missing (Félix, Branco, and Feio, 2013).

As a result, the Global Shelter Cluster is examining pathways towards environmentally‐sustainable assistance, taking both climate change adaptation and mitigation measures (Global Shelter Cluster, 2023). Efforts include advocacy and development of environmental guidelines, standards, and practical assessment tools such as the Nexus Environmental Assessment Tool (NEAT+) and the Shelter Methodology for the Assessment of Carbon (SMAC). Furthermore, lowering the environmental impact has become a strategic priority in the *2024–2028 GSC Strategy* (Global Shelter Cluster, 2024). Owing to limited material expertise and research funding within humanitarian organisations, circularity, retaining material value and lowering GHG emissions, and sustainability of shelter relief products are underexplored. Circularity and sustainability indicators need to be extended to quantify the carbon footprint of humanitarian processes and inform decision‐making, not overlooking variation in humanitarian contexts (Kuittinen, 2016).

This study focuses specifically on the circularity and sustainability of humanitarian emergency hospital shelters, commonly provided as rapid‐assembly modular multipurpose tent structures. Currently, research and information on the procurement and environmental impact of emergency hospital shelters are limited, complicating the evaluation and reduction of GHG emissions for this shelter type. Emergency hospital shelters are often procured and deployed in response to health emergencies, conflicts, or disasters triggered by man‐made or natural hazards, making them an essential part of emergency construction. Because of the specific medical requirements, emergency hospital shelters are mostly managed by non‐governmental organisations (NGOs) that specialise in emergency healthcare, such as Medicines Sans Frontières (MSF) and the International Federation of Red Cross and Red Crescent Societies (IFRC). While ensuring a fully sustainable hospital shelter requires consideration of the planet, people, and prosperity, the scope of this research centres on environmental sustainability and only briefly considers economic and cultural aspects.

The study aims to provide insight into the environmental impact of emergency hospital shelters, identify circular limitations and opportunities, and support procurement decisions. It seeks to answer the following research question: how can the circularity and environmental impact of emergency hospital shelters be assessed and enhanced? Hence, it develops a decision‐support framework to clarify circular and sustainable criteria for emergency hospital shelters. Furthermore, this study evaluates the emergency hospital shelter lifecycle and presents potential improvements for the design, procurement, logistics, use, and end‐of‐life stages. Lastly, several recommendations for strategic, product, and process enhancements in the circularity and sustainability of emergency hospital shelters are provided.

## 2 BACKGROUND

### 2.1 Climate change mitigation in humanitarian shelter

Climate change is one of the greatest health threats facing humanity (Watts et al., 2015; WHO, 2023). Globally, the influence of the climate emergency is observed in all human systems and ecosystems, including water scarcity and food production, cities, and health and well‐being. Regarding the latter, effects such as extreme heat, malnutrition, mental health issues, and displacement are all ‘highly’ or ‘very highly’ associated with climate change, according to a report by the Intergovernmental Panel on Climate Change (Pörtner et al., 2023). There is wide consensus that climate change mitigation is crucial to maintain a world habitable by future generations while simultaneously adapting to unpreventable changes (Pörtner et al., 2022).

Legislation and objectives aimed at mitigating the effects of climate change developed by many policymakers and organisations, such as the European Union (EU) (Baur, 2021)^1^ and the UN (2015a), have led to global climate awareness. For example, the Paris Agreement of 2015 (covering climate change mitigation, adaptation, and finance) tried to establish an international legal framework to remain within two degrees Celsius of global warming (Streck, Keenlyside, and Von Unger, 2016). Additionally, other international goals, such as the SDGs, have been set to reduce GHG emissions and global warming therewith (UN, 2015b). As part of the European Green Deal (2020), the EU states that it seeks to be climate‐neutral by 2050, having an economy with net zero GHG emissions (European Commission, n.d.a). This is to be achieved while also ensuring that ‘no person and no place [are] left behind’ (European Commission, n.d.b). Alongside the SDGs and the Paris Agreement, the Sendai Framework for Disaster Risk Reduction 2015–2030 is calling for sustainable development based on a symbiosis of disaster risk reduction and climate change action (UNDRR, 2021).

The ambitions to address climate change mitigation in humanitarian aid have led humanitarian organisations to develop *The Climate and Environment Charter for Humanitarian Organizations* (ICRC and IFRC, 2021), and resulted in actions by different humanitarian organisations to enhance circularity and sustainability (Joint Initiative for Sustainable Humanitarian Assistance Packaging Waste Management, 2022). We describe those actions most relevant to humanitarian shelter and settlement assistance.

One of the major deployers of humanitarian shelters is the United Nations High Commissioner for Refugees (UNHCR). In the *UNHCR Environmental Guidelines*, the agency states that ‘[e]nvironmental considerations need to be taken into account in almost all aspects of UNHCR's work with refugees and returnees’ (UNHCR, 2005, p. 5). Moreover, regarding shelter construction, the document adds that ‘it is important to ensure the complete availability of appropriate materials, which are either environmentally benign or which have been gathered in a sustainable manner’ (UNHCR, 2005, p. 23). UNHCR published a *Shelter and Sustainability* overview which underscores that the environmental considerations of the type of materials used in shelter design should be integrated as a main planning parameter (Pirjevec, 2021).

Another major deployer of emergency shelters is the IFRC, which recently published *Ambitions to Address the Climate Crisis* (IFRC, 2020). Apart from increasing understanding of the climate crisis, influencing (among other things) investments, policies, and laws, the publication strengthens its expertise in reducing the impact of climate change and in innovating, developing, and enhancing tools. IFRC aims to decrease its own carbon footprint. It will limit its contribution to the climate crisis by ‘measurably reducing [its] carbon footprint and environmental impact’ (IFRC, 2020, p. 10). Moreover, it will seek to ‘ensure that IFRC and ICRC [International Committee of the Red Cross] are leaders in the field of greening their operations’ (IFRC, 2020, p. 10). In 2023, IFRC published a *Green Response: Environmental Quick Guide* (Reynolds et al., 2023) followed by a *Green Response: Green Logistics Guide* (Brangeon, Casagrande, and Galvez, 2023) as steps towards this goal. No specific guidelines have been developed yet that reflect upon the circularity of emergency shelters, let alone emergency hospital shelters.

The largest deployer of emergency hospital shelters, MSF, has set the objective, in *The Environmental* Pact, of reducing its emissions by at least 50 per cent by 2030 as compared to levels in 2019 (MSF, n.d.). It is committed to adapting its activities to reduce significantly its carbon footprint. However, having a sustainable business model has never been one of the core priorities of MSF, which admits that it is ‘rather late to the game of addressing the climate emergency’ (MSF, n.d.).

The importance of context is continuously highlighted in humanitarian assistance, with research stressing that every situation is unique and there are no one‐size‐fits‐all solutions (IFRC and UN OCHA, 2015; Antonini, Boeri, and Giglio, 2020). To lessen the environmental impact, and to respect vernacular and locally‐appropriate solutions, local resource use is advocated in the localisation agenda (Australian Aid et al., 2016). Local resources, though, are still underrepresented among humanitarian hospital shelters in the emergency phase. To avoid dependency on emergency solutions, there is an increasing call for anticipatory action, acting upon predictable risks to reduce vulnerabilities (Anticipatory Action Task Force, 2021).

So far, there is little information on implementing circularity in the humanitarian sector, underlining the need for more research on this topic. One ongoing project is the ‘Waste Management Measuring, Reverse Logistics, Environmentally Sustainable Procurement and Transport, and Circular Economy’ (Logistics Cluster, 2022), developed by the Global Logistics Cluster in collaboration with various humanitarian partners. Under this project there are several guidelines, including *A Circular Economy Introductory Guide* (Logistics Cluster, 2023a), portraying the benefits of circularity for the humanitarian sector. ICRC, UNHCR, and IFRC (2021) have joined forces in the *Eco‐design Tarpaulin Project 2021–2023*, which aims to redesign commonly‐used tarpaulin and thus reduce its environmental impact. ACTED (Agency for Technical Cooperation and Development) developed a circularity toolkit for humanitarian stakeholders under a project titled ‘Circular Economy in the Humanitarian Sector in Jordan’ (Ministry of Environment et al., 2021). There are several guidelines on incorporating sustainable practices in shelter construction, such as the aforementioned *Shelter and Sustainability* overview (Pirjevec, 2021), yet, information on circular shelter practices remains scarce. A notable exception is Gospodinova (2023), who developed an approach for circularity‐informed design decisions for transitional family housing units. This study seeks to assess both circularity and sustainability.

### 2.2 Assessing the circularity and sustainability of humanitarian shelters

Although circularity and sustainability frameworks have overlapping principles, they are not the same. Circularity focuses on closing resource loops and thus retaining material value, whereas sustainability is more broadly associated with maintaining a balance between the planet, people, and prosperity. Nevertheless, both frameworks are important to understand in relation to the humanitarian sector and in particular emergency hospital shelters, as they could improve existing value chains.

One of the key definitions of *sustainability* was presented by the Brundtland Commission: ‘development that meets the needs of the present without compromising the ability of future generations to meet their own needs’ (World Commission on Environment and Development, 1987, p. 37). Recently, *circularity* has gained prominence, and the circular economy has been defined by the Ellen MacArthur Foundation (2021, p. 3) as ‘a systems solution framework that tackles global challenges like climate change, biodiversity loss, waste, and pollution’.

Circularity mostly addresses the needs of future generations by aiming to stop the creation of waste and downcycled materials, as well as to reduce the use of new resources, fight material scarcity, and mitigate climate change by limiting the use of fossil‐based resources (Ellen MacArthur Foundation, 2013). It tries to move away from the linear take–make–waste model towards a regenerative economic system by following the main principles of eliminate waste and pollution, circulate products and materials, and regenerate nature (Ellen MacArthur Foundation, 2013). Via circular principles, components utilised in a humanitarian shelter can become a material source for products with the same or different function (Perrucci and Baroud, 2020). Thus, if implemented properly, there is the potential to lower new product expenses in the shelter sector. Furthermore, circularity can foster innovation in the business models, processes, and products employed in humanitarian construction.

Nevertheless, not all circular practices are automatically environmentally‐friendly. A circular value chain could potentially come along with higher environment‐polluting emissions as compared to a linear alternative (Zink and Geyer, 2017). It is essential, therefore, to calculate and weigh the importance of possible additional emissions versus the circularity benefits. In other industries, tools to measure and compare alternatives are being developed and implemented. For example, ‘The New Normal’ is a circular guideline jointly developed by key stakeholders within the construction sector in the Netherlands (Het Nieuwe Normaal, 2023). With this guideline, the different aspects of the circularity performance of a project can be made transparent, revealing both the emissions and the potential to reuse efficiently materials when the construction is being dismantled. Dismantling buildings to utilise the materials again has also been called *urban mining* (Aldebei and Dombi, 2021). An increasing number of construction projects is putting urban mining into practice (Circl.nl, 2017), although the percentage of mined materials utilised is still far from 100. To assess the circularity and sustainability of humanitarian shelters, criteria need to be compared in a transparent and decisive manner.

## 3 METHODOLOGY

### 3.1 Data collection

This study aims to explore how the circularity and sustainability of emergency hospital shelters can be assessed and enhanced (see Table 1). The research combines a literature study, expert interviews, co‐creation and design sessions, and pilot testing.

**TABLE 1:** Research methodology.

Objectives		Methods
1	Identification and evaluation of sustainability and circularity frameworks and performance indicators	Literature study Nine expert interviews
	Evaluation, transformation, and operationalisation of environmental performance indicators in the design of a decision‐support framework	Three co‐creation workshops, seven key stakeholder interviews
	Pilot testing of the decision‐support tool for sustainable and circular emergency hospital shelters	Fifteen iterative design improvement sessions and pilot testing with practitioners
2	Circularity and sustainability assessment of existing emergency hospital shelters	Qualitative and quantitative assessment
	Identification of guidelines to enhance circularity and sustainability of emergency hospital shelters	One evaluation workshop

**Source:** authors.

The literature study evaluated existing decision‐making models for circular and sustainable product choices and identified a set of performance indicators. It appraised the importance and weighing of environmental‐related sustainability aspects, costs, and the circularity of emergency hospital shelters. It included the environmental burden of raw materials, transportation of products, product lifespan, reuse of materials or components, and end‐of‐life scenarios along with their associated costs. The literature study provided input for the interview questions and the development of an initial assessment approach.

In‐depth semi‐structured expert interviews with six internationally‐operating humanitarian practitioners, two humanitarian shelter suppliers, and one shelter and settlement academic were used to examine current circularity and sustainability assessments in the humanitarian sector, identify performance indicators, and reflect on the relevance of existing frameworks and indicators. Experts were asked to describe common practices, alternatives, decision‐making procedures, preferences, and innovations linked to materials, transport, construction on‐site, and usage and the after‐use phase of emergency hospital shelters (see Appendix A in the supplementary materials).^2^ These scoping interviews painted a good picture of the state of the art, which cannot be found in the literature. Additional interviews were held with seven key stakeholders—shelter procurement and construction personnel of one humanitarian organisation working with emergency hospital shelters—to understand how decisions are currently informed by circularity and sustainability (see Appendix B in the supplementary materials). All interviews were transcribed and analysed.

Three co‐creation sessions concentrated on the evaluation, transformation, and operationalisation of theoretical frameworks and indicators to assess the circularity and sustainability of emergency hospital shelters and to develop a decision‐support tool. An interdisciplinary group of invited stakeholders participated in the workshops, including environmental scientists, business administrators, international shelter experts, and humanitarian shelter and procurement practitioners. In total, 72 different stakeholders took part. The sessions were three months apart and informed continuous optimisation and design iterations, resulting in a well‐informed assessment approach.

Informed by the interviews and co‐creation sessions, the approach was translated into a Microsoft Excel format decision‐support tool and optimised for implementation in approximately 20 weekly design improvement sessions with academic team members or one of the key stakeholders, including shelter and procurement practitioners with a medical humanitarian organisation and an emergency hospital shelter supplier. Appendices D and E in the supplementary materials present the indicators for the assessment.

The assessment tool served to evaluate several emergency hospital shelters using data from suppliers to test the utility of the tool and reflect on existing products and processes. The results were examined by a group of humanitarian procurement practitioners, a supplier, and the academic team.

An interactive in‐person evaluation workshop was organised to appraise the assessment approach and tool. The directors of a research laboratory, a supplier, and the humanitarian organisation participated next to procurement officers, environmental scientists, shelter designers and practitioners, and supply chain and circular economy experts. The workshop identified guidelines for circular and sustainable procurement, logistics, and end of life.

### 3.2 Scope and scale of analysis

**FIGURE 1:**
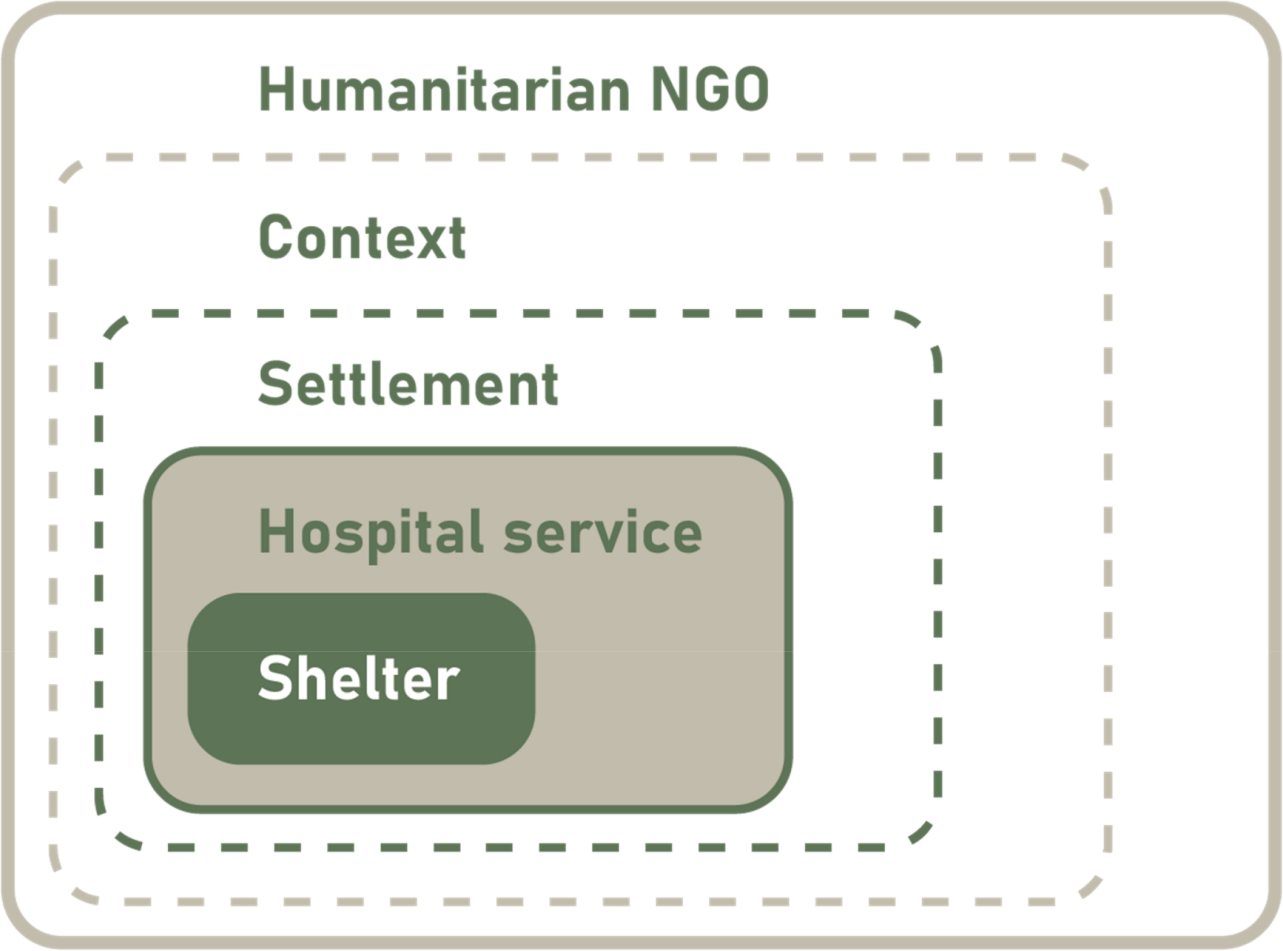
Scope: focus on shelters within the hospital service, settlement, context, and humanitarian aid process **Source:** authors.

NGOs operate under varying conditions of humanitarian missions, with a diversity of cultures, regulations, climates, and emergencies, as displayed in Figure 1. Within or in the proximity of settlements, hospital services are provided. The focus lies on the full lifecycle of the emergency hospital shelter and its components, including the logistics of the shelter *by* the NGO. Production logistics and logistics of distribution *to* the NGO and the wider organisational process of the hospital service are not part of the scope. Therefore, circularity is studied by concentrating on the ‘shelters as a product’ and excludes the full hospital service, business, and economy related to shelter procurement. Similarly, sustainability is studied by considering only the environmental aspects and excluding the social and economic features. To enhance the circularity and sustainability performance of the full hospital service process or humanitarian mission as such, though, the level of analysis of the entire organisation needs to be addressed.

## 4 RESULTS

### 4.1 Developing an assessment approach for circularity and sustainability

Ideally, an assessment uses clear measurable criteria providing unambiguous results. However, circularity and sustainability criteria are not always quantifiable. The carbon footprint of one kilogram of aluminium can be calculated accurately in detail, whereas criteria like possibilities for reuse or refurbishment are often a ‘yes’ or ‘no’, depending on the state of the component. For a combined assessment approach, qualitative and quantitative assessment results need to be balanced and interpreted by the user.

To understand the sustainability of a product, the ReCiPe 2016 method offers a quantitative environmental impact assessment. ReCiPe is a name representing the initials of the developers of the methodology that provides a ‘recipe’ for the calculation of environmental impacts (Ministry of Health, Welfare and Sport, National Institute for Public Health and the Environment, 2024). This method distinguishes 18 different environmental effects (indicators) that can be summarised in three main categories of damages: (i) damage to human health; (ii) damage to ecosystems; and (iii) damage to resource availability. These damages can be presented in the form of carbon emissions. ReCiPe includes indicators related to resource use, but not specifically ones to assess circularity performance.

**FIGURE 2:**
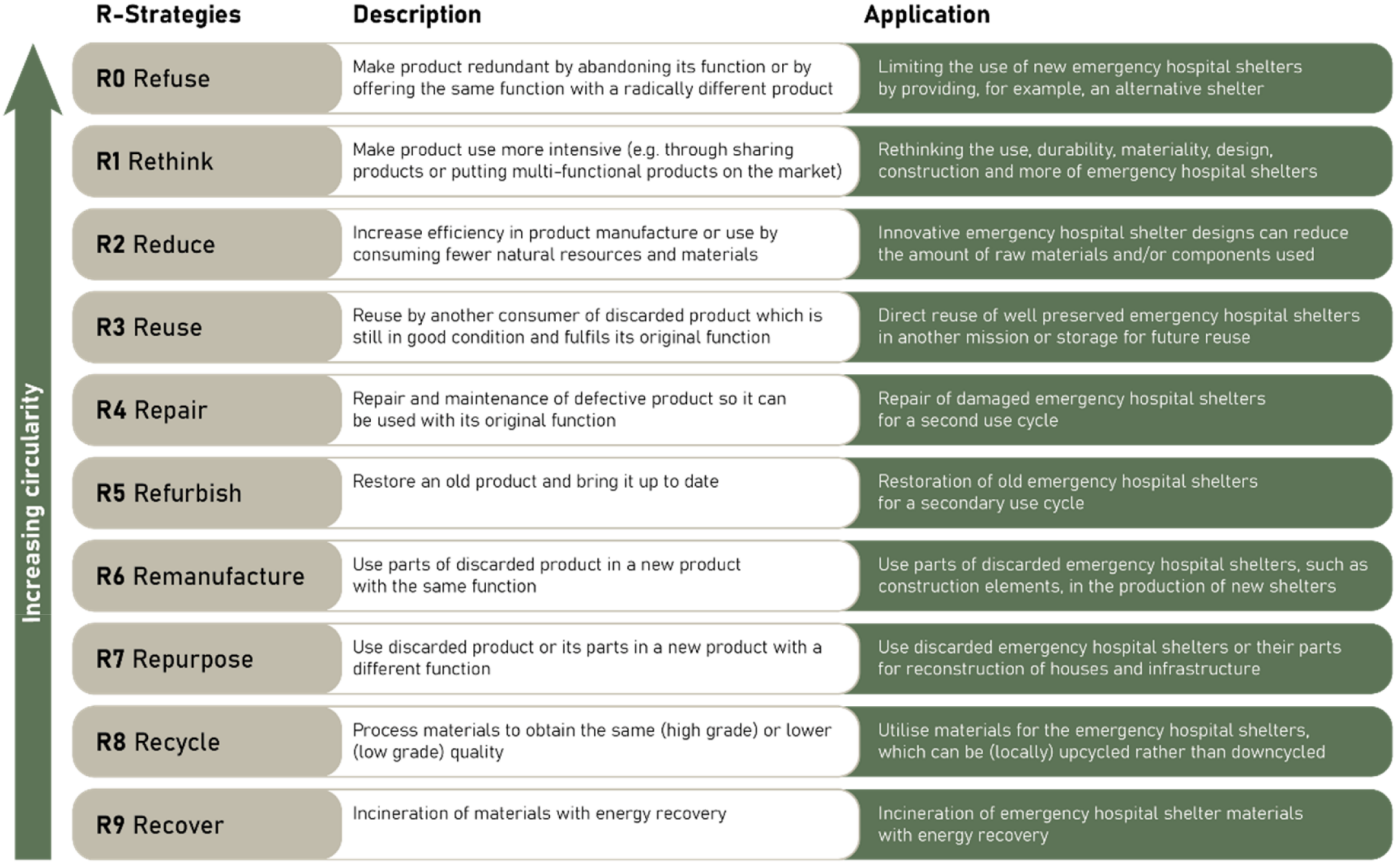
Ten R‐strategies and their theoretical examples of potential application for emergency hospital shelters **Source:** authors, adapted from Potting et al. (2018).

To assess circularity, this study builds upon the circularity strategies defined in the R‐ladder of the Circular Economy Toolkit (Evans and Bocken, n.d.; see Figure 2). Those strategies can be applied in the planning and assessment of humanitarian hospital shelters, starting with the most effective ones. A first step is limiting the use of emergency hospital shelters, questioning if they are necessary in a certain situation or whether they can be ‘refused’ (R0) in specific humanitarian missions, such as by providing an alternative. Another option is ‘rethinking’ (R1) their use, endurance, design, materiality, and more. Yet, in many humanitarian missions, there is no easy alternative to rapidly deployable emergency hospital shelters. Redesign of the product can help to ‘reduce’ (R2) the number of materials used, particularly raw materials. In turn, this may decrease the shelter weight and therefore the transportation costs.

The first strategies aim to improve the product's production and use phases, whereas the following ones seek to prolong the lifecycle of products and their components. After initial utilisation in a mission, well‐preserved emergency hospital shelters can be ‘reused’ (R3) directly by the same or another organisation in another mission or stored for future use. Donation can also be a good alternative, potentially after necessary ‘repair’ (R4) or ‘refurbishment’ (R5). Remaining shelter components can be used for ‘remanufacturing’ (R6) new shelters or ‘repurposed’ (R7) into other products without extensive processing. When all of these strategies are no longer a realistic option, and the product has reached the end of its life as a shelter, some value could potentially still be retained by ‘recycling’ (R8) of the materials. If recycling is not possible, energy can be ‘recovered’ (R9) through incineration. As a last resort, linear options like incineration without energy recovery, landfilling, or burial can be considered, which are not aligned with circularity principles but are not an uncommon fate of shelters today, as the expert interviews and co‐creation sessions revealed.

Although the R‐ladder enables analysis of the circularity of humanitarian emergency hospital shelters, there are no explicit practical tools to make choices between the different R‐strategies in practice. The boundaries between the R‐levels are not simply defined, such as the difference between repair and reuse. How to assess the R‐levels in practice has been explored by others but has not yet been defined for this type of shelter. In addition, the Rs are based on a linear approach but do not necessarily reflect on the overall organisational process, which is more dynamic. What also remains to be defined is at what level products and processes should be analysed, balancing the macro, meso, and micro levels. The current methods describe percentages for water, material use, and energy use as an indication of circularity, but they are not standardised. It is still challenging to assess the percentual impact of the total weight of products.

The R‐ladder strategies can be arranged according to the following criteria: (i) excessive material usage; (ii) biodegradability; (iii) scarce material usage; iv) toxic material usage; (v) lifetime/durability; (vi) cost of repair; (vii) maintenance service; (viii) standardised modules; (ix) second‐hand sales market; (x) cost of refurbishment; (xi) remanufacturing possibilities; (xii) ease of disassembly; (xiii) damage during disassembly; (xiv) possibilities for upgrading the product; (xv) tools needed for disassembly; and (xvi) low material combinations, encased materials.

**FIGURE 3:**
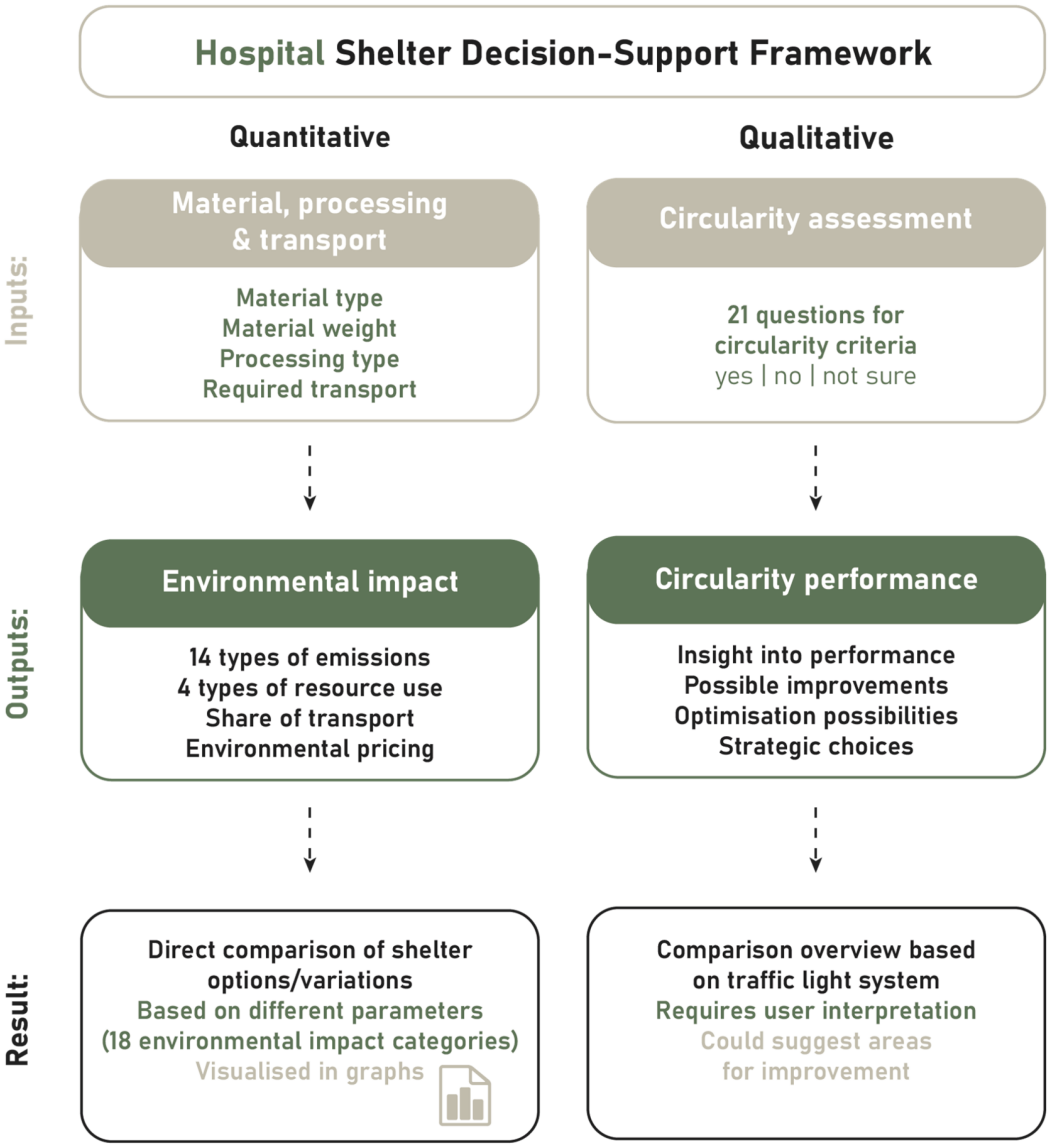
Sustainable and circular shelter decision‐support framework **Source:** authors.

Through several iterations, an assessment framework to support both circular and sustainable decisions is developed. The R‐ladder circularity criteria have a different nature than the criteria used in the ReCiPe method. Therefore, for the decision‐support tool, circularity performance criteria from the R‐ladder can be translated into ‘yes’/‘no’ questions (see Appendix E in the supplementary materials). Environmental sustainability can be assessed using quantifiable ReCiPe 2016 indicators based on the material type, weight, and processing (see Table D1 in Appendix D in the supplementary materials) and the transport choices in the mission (see Table D2 in Appendix D in the supplementary materials). The final version uses ReCiPe 2016 to calculate the estimated *true costs* as defined by CE Delft, an environmental consultant in the Netherlands (de Bruyn et al., 2023). The tool provides several outcomes, allowing for transparent comparison and evaluation of emergency hospital shelters. It represents the scores of various emergency hospital shelter types on different levels of environmental impact and environmental damage cost (Environmental Prices (true costing), Impacts Persons Year Equivalent, Damage to Human Health (DALY), Damage to Ecosystems (species*year), Damage to Resource Availability ($), Fossil Depletion (kg oil eq), Freshwater Consumption (m^3^), Metal Depletion (kg Cu eq), % of Recycled Materials). The circularity performance is visualised in the tool as a ‘traffic light’: ‘yes’ = green, ‘no’ = red, ‘not sure’ = orange (see Figure 3).

The decision‐support tool can be used in the procurement process of the affiliated NGO or as a guideline in the pre‐construction phase, translating priorities and objectives into key performance indicators. Furthermore, it facilitates informed decisions by tactical and operational staff members, displaying contradictory outcomes besides the overall scores. Sometimes criteria are not easily comparable and can be contradictory. As our interviews clearly highlight, not everything is achievable at the same time. It is a dynamic balance between quality standards, immediate delivery, low environmental emissions, and reusing or refurbishing existing components. Organisation leaders need to clarify priorities, to define the operational boundaries at the tactical level. Hence, our tool does not represent one outcome but affords transparency to the user to make informed decisions based on the latest organisational priorities.

### 4.2 Current sustainability and circularity of emergency hospital shelters

Using the assessment criteria, the circularity and sustainability of different emergency hospital shelter components and materials are mapped:

- Emergency hospital shelters consist almost entirely of virgin materials and hardly include any reused, refurbished, or recycled materials. The main construction components contain materials like aluminium and PVC (polyvinyl chloride), which are finite and belong to the ‘technical cycle’ of the circularity butterfly diagram (Ellen MacArthur Foundation, n.d.). Currently, no materials used for the prefabricated shelters would fit with the ‘biological cycle’ of the model, derived from renewable resources. There are opportunities to rethink material use.
- At the start of a mission, an on‐site inventory of the possibilities to use local materials and existing structures for the hospital is commonly produced: ‘You build with what you have, and you work with local materials’ (interview with a shelter practitioner). This inventory is an example of circular practices already present in emergency assistance practice. The procedure aligns with the philosophy of the Sendai Framework to strengthen existing constructions to withstand hazards by ‘building back better’, and simultaneously reduce the need for temporary structures and waste (UNISDR, 2015).
- Data are lacking that reflect on the end‐of‐life scenarios of emergency hospital shelters. More data are needed to paint a complete picture of the circularity and environmental impact.

### 4.3 Mapping end‐of‐life scenarios

To establish a continuous improvement cycle, the existing data gap that inhibits a good understanding of the circularity and sustainability of emergency hospital shelters throughout their lifecycle needs to be addressed. Although data on environmental emissions of the shelters' components and production are relatively easy to obtain, sharing them (as part of the decision‐support tool) is rather difficult owing to information ownership rights. Furthermore, data availability appears more complicated if one searches down the supply chain. After the distribution of emergency hospital shelters, obtaining information becomes very difficult.

There are different reasons for this lack of information. First, the humanitarian accounting system considers a mission to be operating costs; emergency hospital shelters are not regarded as fixed assets. Although this makes perfect accounting sense, it simply means that the shelters disappear from the administrative radar as soon as they are provided on a mission site. The current supply chain, at least from an administrative perspective, is, therefore, a linear system. Second, no data are available on the end‐of‐first‐use phase. Secondary use scenarios are known to respondents, but quantitative data does not exist and are hard to collect because of the extreme workload of field staff.

However, within a humanitarian mission, emergency hospital shelter use can develop from primary to secondary. This is frequently the case when a mission expands in time. In such a situation, the emergency hospital shelter can be upgraded to a semi‐permanent facility by changing modules, such as wall elements. Still, what happens after a mission remains uncertain upfront. The expert interviews revealed that shelters were commonly donated, abandoned, buried, or burned. Yet, no quantitative data are collected mapping the scale of these problematic after‐life scenarios.

### 4.4 Reverse logistics as alternative end of life

Reverse logistics could provide an alternative to the current non‐circular end‐of‐life scenarios. Taking emergency hospital shelters into stock again after a mission could potentially improve their circularity performance, as the introduction of feedback loops in a business process model (see Appendix C in the supplementary materials). This would require a quality check at the end of a mission, to establish whether a shelter is fit for reuse or needs repair, or if it is of no use anymore. Furthermore, if allowed by local government, the different regional warehouses should be equipped to clean and repair shelters and take them back into stock. Additionally, the role of the supplier of the shelters must be evaluated: what part can they play in this future state? Is the supplier ready and willing to take back used emergency hospital shelters and clean, repair, remanufacture, or refurbish the modules? This idea would also necessitate the development or adaptation of the administrative systems of the NGO. How can the emergency hospital shelters be tracked? How is the quality assessment registered? The current situation already shows a data gap. The lack of data and data management is a crucial hurdle to overcome to enhance circularity and sustainability.

One respondent pointed out the potential problems of ‘reverse logistics’. Notably, taking shelters into a country is not the same process as taking them out again. Rules and regulations can differ from country to country and exporting ‘second‐hand’ or used products could be more difficult and expensive than importing new shelters. In some cases, it can even be forbidden to export items because of certain agreements: ‘Sometimes we have an agreement that we don't have to pay duties or VAT [Value Added Tax] anyhow, but then, are we still allowed to export? Sometimes we are not because that means you would be considered a different type of entity, maybe a trading entity’ (interview with a logistics expert).

The concept of ‘reverse logistics’ caused certain unease with the experts during the co‐creation sessions, including: ‘It is really far away from business as usual’. This underlined the necessity of a fundamental change in current practices if one desires to achieve 100 per cent circular practices. Following a step‐by‐step approach would mean that reverse logistics is not the first step to be taken. On a road map, reverse logistics would require an innovation strategy of its own.

### 4.5 Extending and increasing stakeholder responsibilities throughout the product lifecycle

The analysis showed that emergency hospital shelters cannot be treated as simple products. Our examination of emergency hospital shelters revealed that the affiliated humanitarian organisation and supplier had worked with each other for years and had already developed shelters and knowledge together. In the Kraljic Matrix for procurement strategies (Kraljic, 1983), this relationship would classify as a strategic partnership. This insight may imply a shift (away from a procurement assessment) to a relation of interdependence (of co‐makers) in which the assessment of shelter circularity becomes a point of departure in a process of continuous improvement. Acknowledgement of the strategic relationship between the NGO and supplier allows for a long‐term process of improvement of circularity performance.

**FIGURE 4:**
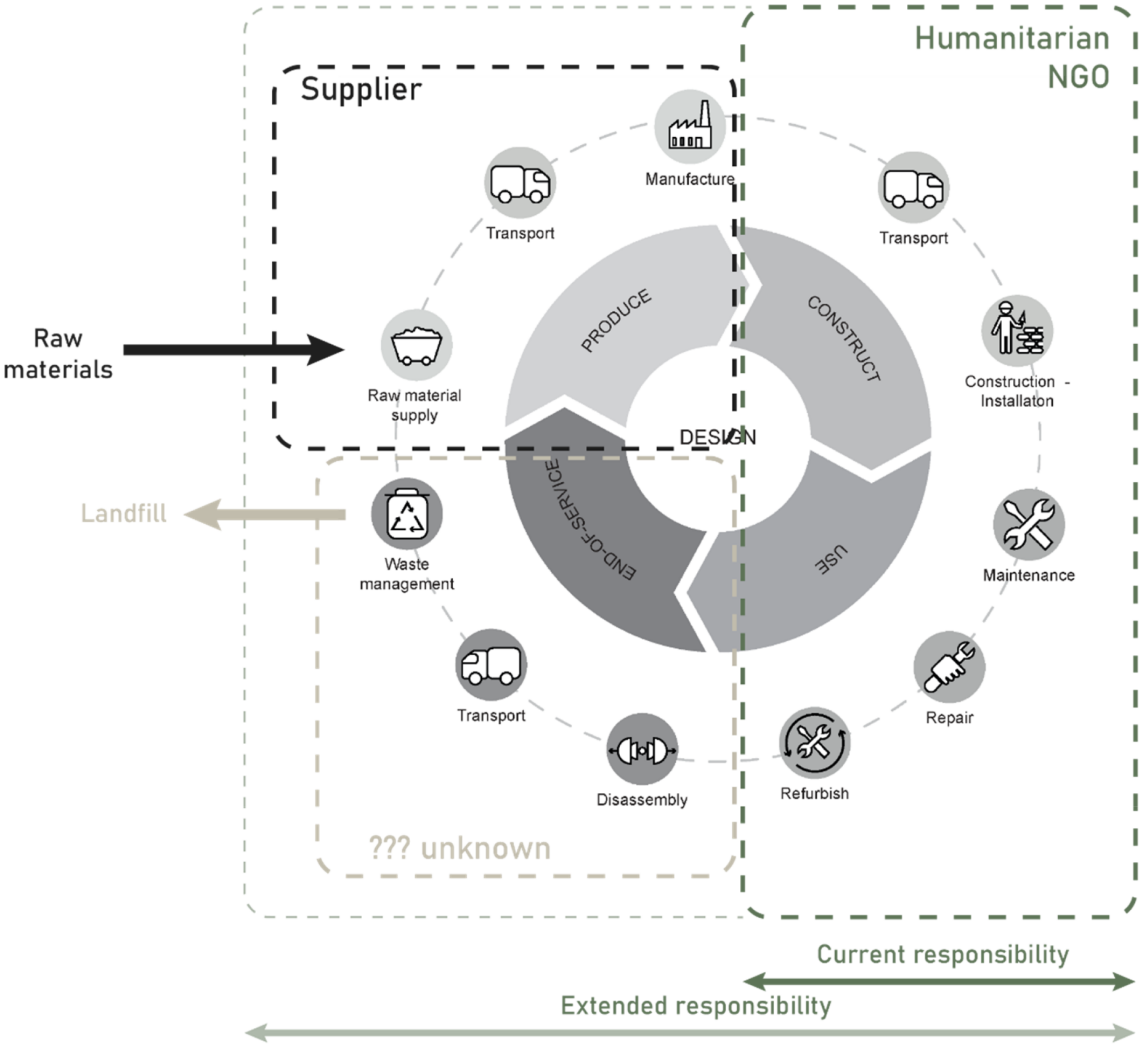
Circularity through extended and increased responsibility in the entire product lifecycle **Source:** authors, adapted from ‘DelftX: Circular Economy for a Sustainable Built Environment’.

The co‐creation sessions demonstrated that stakeholders currently primarily remain within their own boundaries, without considering the entire emergency hospital shelter lifecycle. The responsibility areas of different stakeholders are shown through the dashed lines in Figure 4, adapted from the Circular Product Lifecycle developed by Klein et al. (n.d.). Thus, there is a lack of accountability, while potential links and opportunities to implement R‐strategies are easily overlooked. The end‐of‐service stage especially lacks stakeholder responsibility, including with respect to disassembly, transport, and waste management. Instead, the responsibility of one stakeholder, such as the NGO, can be extended to accountability for the entire lifecycle (the dotted line would simply overarch the entire figure), allowing for novel circular processes. This approach can help to close feedback loops and retain value within the products' system.

The extension of responsibility throughout the lifecycle is a normal phenomenon in literature on circular business models (Geissdoerfer et al., 2020; Lacy, Long, and Spindler, 2020) (for instance, Product as a Service (PaaS) concepts, where the client solely pays for use, while the manufacturer remains the owner), but this responsibility is generally assumed by the supplier or manufacturer, not by the client (in this case the NGO). It would be interesting to investigate further this line of thought. As a starter, it would be good to consider the idea of extending responsibility for a product and the dilemmas that come with that. For example, international NGOs operate in many different contexts, in which it is sometimes impossible to bear responsibility for a shelter, such as when a government simply forbids materials to be taken back out of the country. The complexity of rethinking stakeholder responsibilities aligns with other research (Brügge et al., 2020), stressing that it is by no means self‐evident for humanitarian organisations to implement more sustainable practices. This requires both top‐down vision and support as well as bottom‐up initiatives and willingness to cross beyond the borders of ‘silos’. ^3^

## 5 RECOMMENDATIONS AND DISCUSSION

**FIGURE 5:**
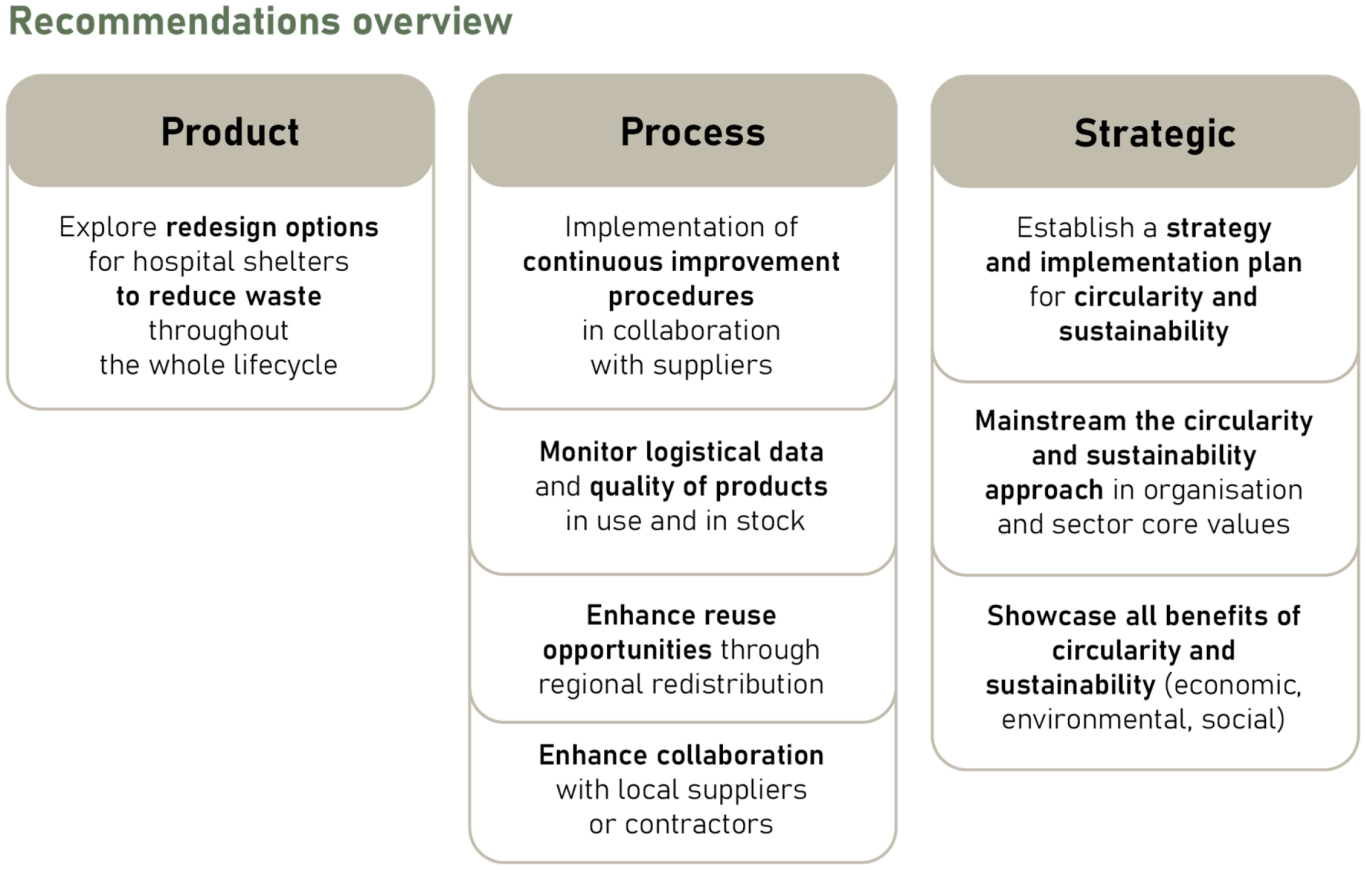
Overview of recommendations **Source:** authors.

The research highlighted several knowledge gaps and obstacles regarding the implementation of circularity and sustainability practices in the sphere of emergency hospital shelters. Based on understanding of existing after‐mission strategies and insights from key stakeholder interviews, several recommendations were developed. These are grouped into product, process, and strategic categories, as presented in Figure 5. While these suggestions have been created particularly for emergency hospital shelters, they could also have a wider implication for other types of shelters.

### 5.1 Product recommendations

Our interview analysis showed that current material use is primarily based on finite materials. We recommend exploring alternative materials that are renewable and/or biodegradable to reduce the environmental impact of both structural and non‐structural components. There are obvious downsides related to transport and durability when aluminium alloys, for example, are replaced with wood or bamboo, or bio‐based plastics instead of PVC sheets. However, innovative solutions might be found in state‐of‐the‐art material developments. We recommend that shelter suppliers and humanitarian organisations actively interact with research laboratories to find fitting material solutions. In addition, strategic partnerships between NGOs and suppliers are key to advancing the circularity of the design and production process.

In addition, our interviews revealed limited developments in the design of hospital shelters, although we see opportunities to reduce waste through smart design, considering the full lifecycle and all circularity strategies. When asked ‘Do you see emergency hospital shelters as circular products? Or “linear” products designed to have an “expiration date”?’, all of our interviewees unanimously demonstrated that emergency hospital shelters are still considered a product with an ‘expiration date’, procured for certain needs and used solely for the project duration. For other items, though, such as laptops, the interviews showed that it is standard practice within the humanitarian sector to consider different ‘after‐life phases’ or ‘exit scenarios’. Shelters, even when in good condition, are commonly not transported back, to avoid freight charges. Emergency hospital shelters are still designed too much as a durable disposable solution, overlooking opportunities for local repair, refurbishing, remanufacturing, repurposing, recycling, and recovery strategies. Developments are needed to explore different hospital shelter designs, typologies, and materials that suit the expected time frame of the emergency mission, varying from three months to 15 years. For instance, additional flexibility in the use of the shelter could support changing functions and allow for longer‐term use. An incremental approach might be suitable for operations that are expected to last longer, while design adaptability can be beneficial for unpredictable crises.

### 5.2 Process recommendations

The research found that systemic change from a linear towards a circular hospital shelter requires collaboration between the different participants in the supply chain. For that reason, a number of recommendations were made that focus on the process of change in the supply chain.

As described in the previous section, the participants in the supply chain of the hospital shelters investigated had a long‐term relationship that had already resulted in innovations that were not specifically circular. To develop a fully circular supply chain, a long‐term process should be implemented, in which partners regularly come together to analyse and improve the supply chain as a whole. In other words, practices of continuous enhancement should be introduced to gradually make the move towards a circular supply chain.

Currently, NGOs do not have the required (logistical) data regarding the (quality of) hospital shelters that are in use or in stock, which are necessary to make decisions about the reuse of those assets. Therefore, alteration of administrative systems is recommended, so that such data can be used to support decision‐making about their reuse.

If data on logistics and the quality of the hospital shelter in use and in stock are available, it becomes possible to improve the reuse of shelters. The results of the study, however, also showed that reuse of hospital shelters often is hindered by regulations that prevent them from being exported again after use. This demonstrates that the processes needed to implement reuse strategies need more changes than just innovation of the supply chain. At the moment, though, no data are available at all, and no steps can be taken.

Circular supply chains in this global field can only be achieved if one improves collaboration with local players as well. Local suppliers and contractors should also be involved in the processes. This is because, first, local actors play an important role in increasing circularity performance. Their products do not need to be shipped and they probably are adapted to local customs. Second, not involving local contributors may spawn a data gap, leaving a part of the hospital facility unmentioned. Third, the use of existing facilities adds up to circularity performance.

### 5.3 Strategic recommendations

We recommend establishing a clear strategy and implementation plan for circularity and sustainability to support difficult procurement decisions. First, who is responsible for the actions to be taken? In line with the findings of Brügge et al. (2020), change has to be initiated and coordinated by the management of humanitarian organisations. It is responsible for the development of the vision on the topic of circularity and sustainability and provides the conditions under which professionals can make responsible decisions. However, because the transition from a linear to a circular humanitarian sector is a profound one, a network like the Global Shelter Cluster may also have a responsibility. For instance, a change of guidelines for emergency hospital shelters may not be left to individual organisations. The Global Shelter Cluster can also provide a platform where representatives of humanitarian organisations can exchange their experiences and cross beyond the borders of their ‘silos’. For a truly circular emergency hospital shelter to become a reality, collaboration between all stakeholders is necessary, as well as a system change in the humanitarian sector.

Circularity and sustainability within humanitarian processes raise ethical dilemmas and calls for value‐oriented discussions. In the humanitarian context, deployment performance and price are constantly balanced. In the current linear system, emergency hospital shelters are seen as a final product, discarding the material value they may possess. A circular alternative is to view the shelter as a resource, thus retaining its value and reducing the need for raw material extraction. A reduction of carbon emissions in the sector can be achieved by financially prioritising and investing in circularity within humanitarian organisations. The difficult debate about the monetary value of circularity and sustainability among humanitarian NGOs and their donors needs to be open, acknowledging that it may decrease the quantity and/or quality of humanitarian aid.

There is also a need for a shift in mindset among practitioners with respect to the fact that there are circular and sustainable opportunities to be explored and considered on a daily basis. We recommend mainstreaming circularity and sustainability approaches in humanitarian organisations and the shelter sector as core values. Right now, the role of the staff members who run the responses in the field is mostly rotative. Thus, by not prioritising shelter circularity within humanitarian organisations, decisions, such as the end‐of‐life scenario, are down to an individual's ethics and values. For that reason, there should be room within humanitarian NGOs for the implementation of a solid ‘circularity and sustainability’ mindset. Incorporating this in the core values and disseminating those ideas to staff are crucial for achieving good results, for example, as a part of the formations and preparations provided to new personnel, who are the people making future crucial decisions in practice.

To support strategic choices and the shift in mindset, we recommend showcasing all of the benefits of circular and sustainable solutions, such as economic, environmental, and social advantages, within the humanitarian organisation, to the assisted population, and to donors. Currently, humanitarian emergency hospital shelters are often designed, procured, produced, and disposed of without a clear understanding of the lasting effects on the local environment or an end‐of‐life strategy. There is potential for stakeholders to learn and reduce their impact.

However, when proposing interventions to align with an ideology of ‘circularity’, one needs to comprehend that the proposed recommendations are not naively going ‘just to work’ and may cause further complications in the existing system of shelter material supply, use, and end of life. Understanding that there are inherent risks of ‘unknowns’ may help to maintain a cautious view of proposals and, at most, be wary of further complications.

### 5.4 Further research

The information gathered here gives an overview of the benefits that circularity practices can bring to the humanitarian sector, regarding, in particular, emergency hospital shelters. The initial steps in this direction, as well as possible limitations, are mapped out. Yet, further research is necessary for a circular emergency hospital shelter to become a reality.

One of the main identified challenges is the lack of numerical data, which indicates the circularity and sustainability status quo of current humanitarian operations. There is limited information on the performance and eventual end‐of‐life routes of emergency hospital shelters. Closing this loop, among others, can contribute to understanding the environmental issues and identifying further knowledge gaps.

There should be a strong focus on ‘end‐of‐life’ research and gathering intelligence through existing experts in the field on what is happening with the materials, and what can be done to introduce circularity at this phase of their lifespan. A goal is that changes can be proposed at various stages of the existing supply change management system of the humanitarian organisation to affect the end‐of‐life phase of the materials. However, the humanitarian sector struggles to monitor the longitudinal impacts of humanitarian interventions covering this stage. The initial humanitarian actors responsible for production, procurement, and deployment are often not present at the end‐of‐life phase owing to the temporary nature of humanitarian work, with constant changes in locations, stakeholders, and political and social dynamics. That makes it difficult to implement tools such as the ones offered by Climate Action Accelerator, which presuppose more or less a certain control by the responsible actor over the lifecycle.

Trying to measure circularity and sustainability accurately is a complex task, resulting in several dilemmas. For example, components made from aluminium or PVC usually have a long lifespan, which is beneficial for environmental performance, but the extraction of the required raw materials and the production of the elements are relatively impactful. Further research is needed to guide shelter designers in their material choices. The decision‐support tool could provide transparency and support informed decisions.

Additional research is needed to pinpoint and implement novel circular and sustainable materials and the use of possible local raw materials for (parts of) the shelter. For instance, the replacement of PVC sheets with bio‐based plastics. Magnesium alloys could potentially replace aluminium frames, learning from large‐scale consumption within the automotive industry. Availability and applicability of bamboo could be explored for the replacement of metal frames and wall infill, owing to its low cost and earthquake‐ and typhoon‐resistant behaviour, as well as local repairability and the potential advantages of local and lightweight transport. However, material changes should also match up with local skills in handling such materials. The effect of material changes on repairs and the carbon emissions of logistics needs to be assessed over the expected lifetime of a shelter.

A lack of openly‐available data was identified as a problem for the decision‐support tool for emergency hospital shelters. Data describing the impact of the extraction of raw materials and the production of shelter components are limited. Where open information is available, it is usually limited to environmental indicators like climate change and water use; the strength of the created tool, though, is to look beyond these two indicators and include 16 other environmental effects which are considered to be equally important. Doing so is what is distinguishing this tool from many others on offer. The tool now includes data which can express the environmental impact of all ReCiPe 2016 indicators, yet this is based on licensed data. Therefore, the tool is not shared or published. Further research is required to investigate the possibilities of using open data for this tool without compromising the strength of being able to assess all ReCiPe 2016 environmental indicators.

Further research is also needed to explore different hospital shelter designs, typologies, and materials that suit the expected time frame of the emergency mission, varying from three months to 15 years. For instance, additional flexibility in the use of the shelter could support changing functions and allow for longer‐term utilisation. An incremental approach might be suitable for operations, which are expected to last longer, while design adaptability can be beneficial for unpredictable crises.

Although logistics are not responsible for the largest share of carbon emissions, research is needed to analyse if local procurement is more sustainable and efficient or if carbon emissions from the current supply chain can be reduced further. An overview of quantities and prices of movements could appraise the viability and trade‐offs of electrification of the supply chain, including the environmental impact and safety of battery use and potential charging and supply disruptions in conflict‐ or disaster‐affected areas. Local procurement could potentially reduce the supply chain if consistent quality and acceptable production standards could be guaranteed for operations. Suppliers could help to identify breakthrough initiatives to make the operations more sustainable. Additionally, a macro‐scale understanding of the systems of shelter material supply and delivery could offer insights into the areas that are within an organisation's responsibility.

Although the steps towards circular emergency hospital shelters seem to call for a radical change in designs and (logistical) processes, this can be achieved only through a long‐term step‐by‐step approach. Stakeholders involved in the production, logistics, and use of the shelters could benefit by implementing continuous improvements in their organisational practices. Research into adopting and maintaining such methods would be a necessary step in any trajectory towards a more circular and sustainable future.

## 6 CONCLUSION

The path towards circular and sustainable emergency hospital shelters remains challenging and complex. This research demonstrates a possibility to assess the circularity and sustainability of existing emergency hospital shelters and identifies several challenges to understanding fully shelter performance.

The study reveals the complexity of circularity and sustainability concepts and evaluations of emergency hospital shelters. Appraising the entire lifecycle of a shelter requires many variables and stakeholders, as well as an extensive amount of data. High circularity potential depends not only on the design of a product, but also is connected to all aspects of an organisation. For a product to be fully circular, all phases—design, material extraction, manufacturing, transportation, construction, use, end of service, and demolition—need to be considered. Despite the challenges ahead, assessing the circularity and sustainability of emergency hospital shelters is a step towards change.

## ETHICS STATEMENT

This paper reports analysis of primary data. The persons from whom data were collected gave their free, prior, and informed consent. Their data have been kept confidential, and the data and analysis have been anonymised.

## FUNDING

This is an independent study, funded through a research grant from the Taskforce for Applied Research SIA for ‘Circular Emergency Shelter’ (project number: KIEM.CIE.06.014) and in‐kind contributions from the Avans University of Applied Sciences, Wijnroemer Relief Goods, and MSF.

Any opinions, findings, and conclusions or recommendations expressed in this study do not necessarily reflect the views of the funding parties.

## SUPPORTING INFORMATION

Additional supporting information may be found online in the Supporting Information section at the end of the article.

## Supporting information


**Data S1** Supporting Information.

## Data Availability

The interview and workshop data that support the findings of this study are available on request from the corresponding author. The data that are now included in the decision‐support tool are licensed data, which cannot be shared. The tool can be shared without data, leaving the framework and principles from which to learn.

## Endnotes

^1^
As of 1 January 2023, the Corporate Sustainability Reporting Directive makes more disclosure of sustainability information by companies mandatory for all of those listed on EU‐regulated markets and for all large companies/groups that meet specific financial requirements (Baur, 2021).

^2^
All supplementary materials can be found online in the Supporting Information section at the end of the article.

^3^
For more information, see Module 3.1.2 (‘Life‐Cycle of a Building’) at https://ocw.tudelft.nl/course-readings/3-1-2-life-cycle-of-a-building/ (last accessed on 12 November 2024). This material was created by or adapted from material posted on the TU Delft OpenCourseware website, ocw.tudelft.nl, and created by Professor Tillmann Klein et al. TU Delft OCW is not responsible for any changes made to the original materials posted on its website and any such changes are the sole responsibility of the authors of this paper. All DelftX courses are under the Creative Commons Attribution‐NonCommercial‐ShareAlike 4.0 International License, https://creativecommons.org/licenses/by-nc-sa/4.0/.

## References

[disa12670-bib-0001] Aldebei, F. and M. Dombi (2021) ‘Mining the built environment: telling the story of urban mining’. Buildings. 11(9). Article number: 388. 10.3390/buildings11090388.

[disa12670-bib-0002] Alshawawreh, L. , F. Pomponi , B. D'Amico , S. Snaddon , and P. Guthrie (2020) ‘Qualifying the sustainability of novel designs and existing solutions for post‐disaster and post‐conflict sheltering’. Sustainability. 12(3). Article number: 890. 10.3390/su12030890.

[disa12670-bib-0003] Anticipatory Action Task Force (2021) Enabling Anticipatory Action at Scale: Policy Brief for Donor Governments . May. https://www.anticipation-hub.org/download/file-1413 (last accessed on 4 November 2024).

[disa12670-bib-0004] Antonini, E. , A. Boeri , and F. Giglio (2020) ‘Technologies for building after disaster: a critical review’. In E. Antonini , A. Boeri , and F. Giglio (eds.) Emergency Driven Innovation: Low Tech Buildings and Circular Design. Innovation, Technology, and Knowledge Management. Springer, Cham. pp. 27–58.

[disa12670-bib-0005] Australian Aid et al. (2016) The Grand Bargain – A Shared Commitment to Better Serve People in Need . 23 May. https://interagencystandingcommittee.org/sites/default/files/migrated/2017‐02/grand_bargain_final_22_may_final‐2_0.pdf (last accessed on 4 November 2024).

[disa12670-bib-0006] Baur, D. (2021) ‘Corporate Sustainability Reporting Directive – April 2021’. PwC (PricewaterhouseCoopers) Switzerland website. 4 May. https://www.pwc.ch/en/insights/accounting/corporate-sustainability-reporting-directive-april-2021.html (last accessed on 12 November 2024)

[disa12670-bib-0007] Brangeon, S. , R. Casagrande , and J. Galvez (2023) Green Response: Green Logistics Guide. International Federation of Red Cross and Red Crescent Societies, Geneva.

[disa12670-bib-0008] Brangeon, S. and F. Crowley (2020) Environmental Footprint of Humanitarian Assistance: Funded by DG ECHO: Scoping Review. Research Paper. May. INSPIRE Consortium, Plaisians.

[disa12670-bib-0009] Brügge, C. , J. Pinochet , S. Hansen , and V. Vichitlekarn (2020) Environmental Mainstreaming in Humanitarian Interventions. London School of Economics and Political Science, London.

[disa12670-bib-0010] Circl.nl (2017) ‘The making of Circl: the story of a “circular” pavilion in Amsterdam's Zuidas district’. Website. September. https://circl.nl/themakingof/en/ (last accessed on 4 November 2024).

[disa12670-bib-0011] de Bruyn, S. et al. (2023) Handboek Milieuprijzen 2023: Methodische onderbouwing van kengetallen gebruikt voor waardering van emissies en milieu‐impacts. February. CE Delft, Delft.

[disa12670-bib-0012] Development Initiatives (2023) Global Humanitarian Assistance Report 2023. Development Initiatives, Bristol.

[disa12670-bib-0013] Ellen MacArthur Foundation (n.d.) ‘What is a circular economy?’. Website. https://ellenmacarthurfoundation.org/topics/circular-economy-introduction/overview (last accessed on 4 November 2024).

[disa12670-bib-0014] Ellen MacArthur Foundation (2013) Towards the Circular Economy Volume 2: Opportunities for the Consumer Goods Sector. Ellen MacArthur Foundation, Cowes, Isle of Wight.

[disa12670-bib-0015] Ellen MacArthur Foundation (2021) Circular Economy Glossary. Ellen MacArthur Foundation, Cowes, Isle of Wight.

[disa12670-bib-0016] European Commission (n.d.a.) ‘The European Green Deal: a growth strategy that protects the climate’. Website. https://ec.europa.eu/stories/european-green-deal/ (last accessed on 12 November 2024).

[disa12670-bib-0017] European Commission (n.d.b.) ‘The European Green Deal: striving to be the first climate‐neutral continent’. Website. https://commission.europa.eu/strategy-and-policy/priorities-2019-2024/european-green-deal_en (last accessed on 12 November 2024).

[disa12670-bib-0018] Evans, J. and N. Bocken (n.d.) ‘Circular Economy Toolkit; resources for an evolving world’. Circular Economy Toolkit website. http://circulareconomytoolkit.org/about.html (last accessed on 4 November 2024).

[disa12670-bib-0019] Félix, D. , J.M. Branco , and A. Feio (2013) ‘Temporary housing after disasters: a state of the art survey’. Habitat International. 40 (October). pp. 136–141.

[disa12670-bib-0020] Geissdoerfer, M. , M.P.P. Pieroni , D.C.A. Pigosso , and K. Soufani (2020) ‘Circular business models: a review’. Journal of Cleaner Production. 277 (December). Article number: 123741. 10.1016/j.jclepro.2020.123741.

[disa12670-bib-0021] Global Shelter Cluster (2023) Greening the Shelter Response . April. https://sheltercluster.org/environment-community-practice/documents/gsc-environmental-activities-2023 (last accessed on 4 November 2024).

[disa12670-bib-0022] Global Shelter Cluster (2024) 2024–2028 GSC Strategy. Soft launch version. June. https://sheltercluster.org/global-strategic-advisory-group/pages/gsc-strategy-2024-2028 (last accessed on 4 November 2024).

[disa12670-bib-0023] Godefroy, B. et al. (2023) A Path to Climate‐Smart Humanitarian Action: Roadmap for Halving Emissions in the Humanitarian Sector by 2023. June. Climate Action Accelerator, Geneva.

[disa12670-bib-0024] Gospodinova, J. (2023) Circular Transitional Housing for Displaced People in Extreme Conditions: The Case of Pakistan. Master's Thesis. Faculty of Architecture and the Built Environment, Delft University of Technology, Delft. https://repository.tudelft.nl/record/uuid:1235cbd2-0170-4780-bf17-a508c4076dde (last accessed on 4 November 2024).

[disa12670-bib-0025] Hald, K.S. , S. Wiik , and A. Larssen (2021) ‘Sustainable procurement initiatives and their risk‐related costs: a framework and a case study application’. Measuring Business Excellence. 25(2). pp. 230–243.

[disa12670-bib-0026] Het Nieuwe Normaal (2023) Leidraad HNN Gebouw: Een eenduidige taal met haalbare én ambitieuze circulaire prestaties voor de bouwsector . Version 1.0. December. https://www.hetnieuwenormaal.nl/leidraden/gebouw/uitgangspunten/ (last accessed on 4 November 2024).

[disa12670-bib-0027] ICRC (International Committee of the Red Cross) and IFRC (International Federation of Red Cross and Red Crescent Societies) (2021) The Climate and Environment Charter for Humanitarian Organizations . https://www.climate-charter.org/ (last accessed on 4 November 2024).

[disa12670-bib-0028] ICRC, UNHCR, and IFRC (2021) ICRC/IFRC/UNHCR Eco‐design Tarpaulin Project 2021–2023 . May. https://logcluster.org/en/document/icrcifrcunhcr-eco-design-tarpaulin-project-2021-2023 (last accessed on 4 November 2024).

[disa12670-bib-0029] IFRC (International Federation of Red Cross and Red Crescent Societies) (2020) Ambitions to Address the Climate Crisis. IFRC, Geneva.

[disa12670-bib-0030] IFRC (International Federation of Red Cross and Red Crescent Societies) and UN OCHA (United Nations Office for the Coordination of Humanitarian Affairs) (2015) Shelter After Disaster. Second edition. IFRC, Geneva.

[disa12670-bib-0031] Joint Initiative for Sustainable Humanitarian Assistance Packaging Waste Management (2022) ‘ Who's Doing What’ on Sustainable Procurement: An Overview of What Humanitarian Organizations Are Doing to ‘Green’ Their Procurement Practices . December. https://eecentre.org/wp-content/uploads/2022/12/Whos-Doing-What-on-Sustainable-Procurement.pdf (last accessed on 4 November 2024).

[disa12670-bib-0032] Klein, T. et al. (n.d.) Circular Economy for a Sustainable Built Environment . TU Delft Online Learning. https://online-learning.tudelft.nl/courses/circular-economy-for-a-sustainable-built-environment/ (last accessed on 4 November 2024).

[disa12670-bib-0033] Kraljic, P. (1983) ‘Purchasing must become supply management’. *Harvard Business Review website*. September. https://hbr.org/1983/09/purchasing-must-become-supply-management (last accessed on 4 November 2024).

[disa12670-bib-0034] Kuittinen, M. (2016) Carbon Footprinting in Humanitarian Construction: What Are the CO_2_ Emissions and How to Mitigate Them? Doctoral Dissertations 51/2016. School of Arts, Design and Architecture, Aalto University, Helsinki.

[disa12670-bib-0035] Lacy, P. , J. Long , and W. Spindler (2020) The Circular Economy Handbook: Realizing the Circular Advantage. Palgrave Macmillan, London.

[disa12670-bib-0036] Logistics Cluster (2022) ‘WREC: about the project’. Website. 13 October. https://logcluster.org/document/wrec-about-project (last accessed on 4 November 2024).

[disa12670-bib-0037] Logistics Cluster (2023a) WREC Quick Guide: A Circular Economy Introductory Guide . May. https://logcluster.org/en/document/wrec-quick-guide-circular-economy-introductory-guide (last accessed on 4 November 2024).

[disa12670-bib-0038] Logistics Cluster (2023b) WREC Quick Guide: Environmentally Sustainable Procurement . July. https://logcluster.org/en/document/wrec-quick-guide-environmentally-sustainable-procurement-july-2023 (last accessed on 4 November 2024).

[disa12670-bib-0039] Ministry of Environment, ACTED (Agency for Technical Cooperation and Development), IMPACT, and United Nations Environment Programme (2021) Circular Economy in the Humanitarian Sector in Jordan . December. https://www.acted.org/wp-content/uploads/2018/01/1.-jordan-circular-economy-humanitarian-summary-vf.pdf (last accessed on 4 November 2024).

[disa12670-bib-0040] Ministry of Health, Welfare and Sport, National Institute for Public Health and the Environment (2024) LCIA: The ReCiPe Model . Website. 29 October. https://www.rivm.nl/en/life-cycle-assessment-lca/recipe (last accessed on 4 November 2024).

[disa12670-bib-0041] MSF (Medicines Sans Frontières) (n.d.) ‘Climate emergency’. Website. https://www.msf.org/climate-emergency (last accessed on 4 November 2024).

[disa12670-bib-0042] Perrucci, D. and H. Baroud (2020) ‘A review of temporary housing management modeling: trends in design strategies, optimization models, and decision‐making methods’. Sustainability. 12(24). Article number: 10388. 10.3390/su122410388.

[disa12670-bib-0043] Pirjevec, A. (2021) Shelter and Sustainability: A Technical and Environmental Comparative Overview of Common Shelter Typologies Found in Settlements Across UNHCR Operations. UNHCR, Geneva.

[disa12670-bib-0044] Pörtner, H‐O. et al. (2023) *Climate Change 2022: Impacts, Adaptation and Vulnerability* . Working Group II Contribution to the Sixth Assessment Report of the Intergovernmental Panel on Climate Change. Cambridge University Press, Cambridge.

[disa12670-bib-0045] Potting, J. et al. (2018) Circular Economy: What We Want to Know and Can Measure. Framework and Baseline Assessment for Monitoring the Progress of the Circular Economy in the Netherlands. Policy Report No. 3217. July. PBL Netherlands Environmental Assessment Agency, The Hague.

[disa12670-bib-0046] Reynolds, G. , R. Casagrande , S. Ritthammer , and J. Sandberg (2022) Green Response: Environmental Quick Guide. International Federation of Red Cross and Red Crescent Societies, Geneva.

[disa12670-bib-0047] Sleeswijk, A.W. , L.F.C.M. van Oers , J.B. Guinée , J. Struijs , and M.A.J. Huijbregts (2008) ‘Normalisation in product life cycle assessment: an LCA of the global and European economic systems in the year 2000’. Science of The Total Environment. 390(1). pp. 227–240. 17996278 10.1016/j.scitotenv.2007.09.040

[disa12670-bib-0048] Streck, C. , P. Keenlyside , and M. Von Unger (2016) ‘The Paris Agreement: a new beginning’. Journal for European Environmental & Planning Law. 13(1). pp. 3–29.

[disa12670-bib-0049] UN (United Nations) (1991) Strengthening of the Coordination of Humanitarian Emergency Assistance of the United Nations: Resolution/Adopted by the General Assembly . A/RES/46/182. 19 December. https://digitallibrary.un.org/record/135197?ln=en&v=pdf (last accessed on 4 November 2024).

[disa12670-bib-0050] UN (2004) Strengthening of the Coordination of Emergency Humanitarian Assistance of the United Nations . Resolution adopted by the General Assembly on 17 December 2003. A/RES/58/114. 5 February. https://documents.un.org/doc/undoc/gen/n03/501/42/pdf/n0350142.pdf (last accessed on 12 November 2024)

[disa12670-bib-0051] UN (2015a) Transforming Our World: The 2030 Agenda for Sustainable Development . Resolution adopted by the General Assembly on 25 September 2015. A/RES/70/1. 21 October. https://documents.un.org/doc/undoc/gen/n15/291/89/pdf/n1529189.pdf (last accessed on 12 November 2024).

[disa12670-bib-0052] UN (2015b) Transforming Our World: The 2030 Agenda for Sustainable Development . A/RES/70/1. https://sdgs.un.org/publications/transforming-our-world-2030-agenda-sustainable-development-17981 (last accessed on 12 November 2024)

[disa12670-bib-0053] UNDRR (United Nations Office for Disaster Risk Reduction) (2021) Promoting Synergy and Alignment: Between Climate Change Adaptation and Disaster Risk Reduction in the Context of National Adaptation Plans. A Supplement to the UNFCCC NAP Technical Guidelines. UNDRR, Geneva.

[disa12670-bib-0054] UNHCR (United Nations High Commissioner for Refugees) (2005) UNHCR Environmental Guidelines. August. UNHCR, Geneva.

[disa12670-bib-0055] UNHCR (2015) UNHCR Emergency Handbook. UNHCR, Geneva.

[disa12670-bib-0056] UNISDR (United Nations Office for Disaster Reduction) (2015) Sendai Framework for Disaster Risk Reduction 2015–2030. UNISDR, Geneva.

[disa12670-bib-0057] UN OCHA (United Nations Office for the Coordination of Humanitarian Affairs) (2021) Global Humanitarian Overview 2022. December. UN OCHA, Geneva.

[disa12670-bib-0058] Watts, N. et al. (2015) ‘Health and climate change: policy responses to protect public health’. The Lancet. 386(10006). pp. 1861–1914. 10.1016/S0140-6736(15)60854-6 26111439

[disa12670-bib-0059] WHO (World Health Organization) (2023) ‘Climate change’. Website. 12 October. https://www.who.int/news-room/fact-sheets/detail/climate-change-and-health (last accessed 12 November 2024).

[disa12670-bib-0060] World Commission on Environment and Development (1987) Report of the World Commission on Environment and Development: Our Common Future. United Nations, New York City, NY.

[disa12670-bib-0061] Zink, T. and R. Geyer (2017) ‘Circular economy rebound’. Journal of Industrial Ecology. 21(3). pp. 593–602. 10.1111/jiec.12506 PMC13068685 41971412

