# Supplementary material for: Assessing and enhancing the circularity and sustainability of emergency hospital shelters
Source: Disasters. 2024 Dec 4;49(1):e12670. doi: 10.1111/disa.12670 (PMC11616682; doi:10.1111/disa.12670)
Supplement: Supplementary file 1 — Data S1 Supporting Information. [file DISA-49-e12670-s001.docx]

**SUPPLEMENTARY MATERIALS**

*Title: Assessing and enhancing circularity of humanitarian shelters*

**Appendix A. Semi-structured expert interview guide**

|  | **Shelter materials** | **Transport** | **Building on-site** | **Usage / After-life** |
| --- | --- | --- | --- | --- |
| **Alternatives** | What materials options do you consider if you look at funding, production, time and sustainability? | What transport options do you consider if you look at funding, production, time and sustainability? | What options are there for the construction process and how is that linked to the size, type of event and country? | What options are there for the tents at the end of a mission? |
| **Common practices** | What are common materials and how are they selected based on size, duration and type of mission and transport options? | What are common transport modes used in your company based on size, duration and type of mission? | What are common construction processes on location? | What are common destinations or treatments of tent structures after a mission? |
| **Criteria for decision making** | With what knowledge or data are materials chosen and justified? | With what knowledge or data is the transport mode chosen and justified? | With what knowledge or data is the construction process chosen or designed and justified? | With what knowledge or data is the destination or treatment of the tent structures chosen after a mission? |
| **Personal preferences** | What materials would you prefer if you do not have to consider environmental impact, funding and time? | What transport would you like to use if you do not have to consider environmental impact, funding and time? | What construction process would you prefer if you do not have to consider environmental impact, funding and time? | What destination or treatment of the tent structures would you prefer if you do not have to consider environmental impact, funding and time? |
| **Innovation** | Are you aware of any innovative recently developed materials? | Are you aware of any innovative transport options? | Are you aware of innovative construction tools or processes? | Are you aware of new or innovative after-life solutions for tents? |

**Appendix B. Semi-structured key stakeholder interviews**

1. **General**

[introduction of research aims]

- Do you have questions about the aims of this study – Is everything clear?
- Do we have your consent to use this interview for academic purposes and *humanitarian organisation X*?
- Could you please introduce yourself?
- What do you think is the difference between sustainability and circularity?
- Do you see emergency hospital shelters as circular products or as linear products designed to have an ‘*expiration date’*?
- In the life cycle phase of an emergency hospital shelter where you operate, are decisions made based on circularity principles?

1. **After-mission**

- What are the possible after-life scenarios for emergency shelters after a mission?
  - Donate? Burn? Sell? Disassemble? Fly to local warehouse? Fly to next mission?
- What scenario is most common? And which one is the least common?
  - Could you give (estimated) percentages?
- Who takes decisions about what happens to emergency hospital shelters after a mission?
- Based on what are these decisions taken about the destination or treatment of emergency hospital shelters after a mission?
- Are there any protocols on what to do with emergency hospital shelters after a mission?
- During a mission, when do you start thinking about an ‘*exit scenario’*?
  - How are the emergency hospital shelters considered in exit scenarios?
- What is the most sustainable option after a mission for emergency hospital shelters according to you?
  - Are there any practical objections/complicating factors that make this difficult to realize?

1. **After-life**

- And what happens when an emergency hospital shelter is not usable anymore?
  - Are they being *repaired, refurbished, remanufactured, repurposed, or recycled*?
  - Are you familiar with R10-model?
- What scenario is most common? And which one is the least common?
  - Could you give (estimated) percentages?
- Who takes decisions about what happens to emergency hospital shelters after-life?
- Based on what are these decisions taken?
- Are there any protocols on what to do with emergency hospital shelters when they are unusable?
- What is the most sustainable option for the after-life of emergency hospital shelters according to you?
  - Are there any practical objections/complicating factors that make this difficult to realize?

1. **General**

- In your opinion, could sustainability and/or circularity of humanitarian missions increase at the expense of (money for) the medical aid provided? Why (not)?
- Which topic did we overlook in our questions and needs our attention?
- Do you have any questions for us? / Is there anything else you would like to address?
- Do you have any contacts that could be useful for this study?

**Appendix C. Business process model**


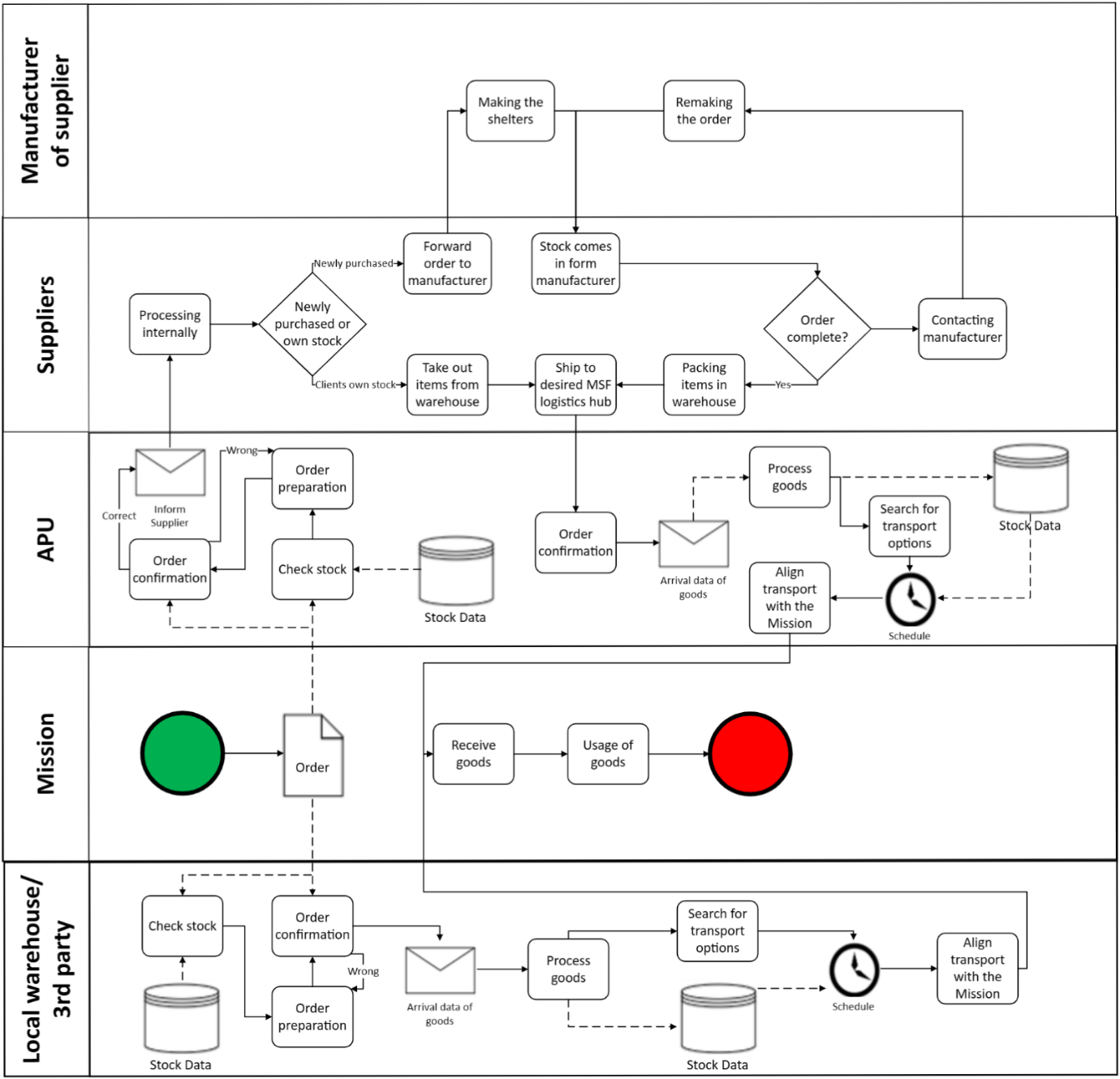


APU = Amsterdam Procurement Unit

**Appendix D. Environmental impact assessment of emergency hospital shelters**

Based on the material type, weight, and processing steps (D1), and transport choices (D2) carbon emissions are calculated. Environmental impact calculations use the input and a paid dataset. Graphs with total carbon emissions are presented in different forms for each shelter separately to allow for comparison: Environmental prices (true costing), Impacts Persons Year Equivalent, Damage to Human Health (DALY), Damage to Ecosystems (species*year), Damage to Resource Availability ($), Fossil depletion (kg oil eq), Freshwater Consumption (m^3^), Metal depletion (kg Cu eq), % of Recycled Materials. Transport emissions are also presented as part of the overall emissions.

D1. Shelter environmental impact assessment based on material choices by supplier

| **Supplier name:** |  |  |  |  |
| --- | --- | --- | --- | --- |
|  |  |  |  |  |
| **Shelter dimensions:** |  | Area: |  | m2 |
| **Total shipping weight:** |  | kg |  |  |
|  |  |  |  |  |
| **Supplier data date:** |  |  |  |  |
| **Revision date:** |  |  |  |  |
|  |  |  |  |  |
| **Price** |  |  |  |  |
|  |  |  |  |  |
|  |  |  |  |  |
| **1** | **Frame** |  | **Weight** |  |
| 1.1 | Material #1 |  |  | Kg |
| 1.1.1 | Processing #1 |  | 103 | Kg |
| 1.1.2 | Processing #2 |  | 103 | Kg |
|  |  |  |  |  |
| 1.2 | Material #2 |  |  | Kg |
| 1.2.1 | Processing #1 |  | 181 | Kg |
| 1.2.2 | Processing #2 |  | 181 | Kg |
|  |  |  |  |  |
| **2** | **Tarp** |  |  |  |
| 2.1 | Material #1 |  |  | Kg |
| 2.1.1 | Processing #1 |  | 75 | Kg |
| 2.1.2 | Processing #2 |  | 75 | Kg |
|  |  |  |  |  |
| 2.2 | Material #2 |  |  | Kg |
| 2.2.1 | Processing #1 |  | 0 | Kg |
| 2.2.2 | Processing #2 |  | 0 | Kg |
|  |  |  |  |  |
| **3** | **Connectors** |  |  |  |
| 3.1 | Material #1 |  |  | Kg |
| 3.1.1 | Processing #1 |  | 0 | Kg |
| 3.1.2 | Processing #2 |  | 0 | Kg |
|  |  |  |  |  |
| 3.2 | Material #2 |  |  | Kg |
| 3.2.1 | Processing #1 |  | 0 | Kg |
| 3.2.2 | Processing #2 |  | 0 | Kg |
|  |  |  |  |  |
| **4** | **Expansion** |  |  |  |
| 4.1 | Material #1 |  |  | Kg |
| 4.1.1 | Processing #1 |  | 0 | Kg |
| 4.1.2 | Processing #2 |  | 0 | Kg |
|  |  |  |  |  |
| 4.2 | Material #2 |  |  | Kg |
| 4.2.1 | Processing #1 |  | 0 | Kg |
| 4.2.2 | Processing #2 |  | 0 | Kg |
|  |  |  |  |  |
| **5** | **Expansion** |  |  |  |
| 5.1 | Material #1 |  |  | Kg |
| 5.1.1 | Processing #1 |  | 0 | Kg |
| 5.1.2 | Processing #2 |  | 0 | Kg |
|  |  |  |  |  |
| 5.2 | Material #2 |  |  | Kg |
| 5.2.1 | Processing #1 |  | 0 | Kg |
| 5.2.2 | Processing #2 |  | 0 | Kg |

D2. Shelter environmental impact assessment based on transport choices in humanitarian mission

| **Transportation lines** | **Transportation type** | **Distance traveled (km)** |
| --- | --- | --- |
| Transport #1  (Supplier to stock) | *None / Truck Euro 0-6 mix / Truck Euro 4 / Truck Euro 5 / Truck Euro 6 / Landcruiser 4x4 / Plane 113t payload / Plane 65 payload / Plane 22 payload / Containership (Ocean)* |  |
| Transport #2  (Stock to project country (port/airport) | *None / Truck Euro 0-6 mix / Truck Euro 4 / Truck Euro 5 / Truck Euro 6 / Landcruiser 4x4 / Plane 113t payload / Plane 65 payload / Plane 22 payload / Containership (Ocean)* |  |
| Transport #3  (Project country (port/airport to location) | *None / Truck Euro 0-6 mix / Truck Euro 4 / Truck Euro 5 / Truck Euro 6 / Landcruiser 4x4 / Plane 113t payload / Plane 65 payload / Plane 22 payload / Containership (Ocean)* |  |
| **Total distance** |  |  |

**Appendix E. Circularity performance of emergency hospital shelters**

This qualitative assessment is based on the R-ladder and translates the circularity strategies into criteria that can be assessed during the procurement phase of a shelter.

|  | **Criteria** | **Questions** | **Answers*** |
| --- | --- | --- | --- |
| R0 | Refuse new materials | Do you need a new shelter? | *Yes/ No / Not sure* |
|  | Refuse new materials | Is there a shelter already in local storage? | *Yes/ No / Not sure* |
| R2 | Reduce for circularity | Is there a high waste of materials during production? | *Yes/ No / Not sure* |
|  | Reduce for circularity | Does the shelter have biodegradable materials? | *Yes/ No / Not sure* |
|  | Reduce for circularity | Does the shelter use scarce materials? | *Yes/ No / Not sure* |
|  | Reduce for circularity | Does the shelter have a longer lifetime than use phase? | *Yes/ No / Not sure* |
|  | Reduce for circularity | Does the shelter use toxic materials? | *Yes/ No / Not sure* |
| R3 | Reuse the shelter | Is the shelter donated? | *Yes/ No / Not sure* |
|  | Reuse the shelter | Does the shelter have a long lifetime? >10 years | *Yes/ No / Not sure* |
| R4 | Repair existing shelter | Is the shelter expensive to repair? | *Yes/ No / Not sure* |
|  | Repair existing shelter | Does the shelter have a maintenance service? | *Yes/ No / Not sure* |
|  | Repair existing shelter | Are the components standardized? | *Yes/ No / Not sure* |
| R5 | Refurbish at the end of life | Is it expensive to refurbish the shelter? | *Yes/ No / Not sure* |
|  | Refurbish at the end of life | Can the shelters be returned and refurbished by the supplier? | *Yes/ No / Not sure* |
| R6 | Remanufacture if you cannot refurbish | Is it difficult to disassemble? (i.e uses weld or glue) | *Yes/ No / Not sure* |
|  | Remanufacture if you cannot refurbish | Is the shelter damaged if disassembled | *Yes/ No / Not sure* |
|  | Remanufacture if you cannot refurbish | Are the parts modular allowing switch in switch out? | *Yes/ No / Not sure* |
|  | Remanufacture if you cannot refurbish | Can the parts be upgraded? | *Yes/ No / Not sure* |
|  | Remanufacture if you cannot refurbish | Are specialized tools needed to disassemble? | *Yes/ No / Not sure* |
| R8 | Recycle if lifespan cannot be extended | Are material combination used in the shelter? (PVC) | *Yes/ No / Not sure* |
|  | Recycle if lifespan cannot be extended | Are there incased materials (i.e plastic around steel) | *Yes/ No / Not sure* |

*R0= refuse, R2= reduce, R3= reuse, R4=repair, R5= refurbish, R6= remanufacture, R8=recycle.*

*Results are presented with colour codes. (Yes=green, No=red, Not sure=orange)
